# Mapping Practice-Based Signals of Generative AI in Psychiatric Care: Qualitative Study of Korean Psychiatrists’ Experiences, Interpretations, and Implementation Priorities

**DOI:** 10.2196/96556

**Published:** 2026-06-02

**Authors:** Myungsung Kim, Yoosuk An, Min Jeon, Yunji Lee, Orane Farrah Lahcine, Hyorim Kim, Seonmi Lee, Sangil Lee, Jong-Chul Yang, Sang-Won Jeon, Dooyoung Jung, Chul-Hyun Cho

**Affiliations:** 1 Graduate School of Health Science and Technology, Ulsan National Institute of Science and Technology, Ulsan, Republic of Korea; 2 Department of Psychiatry, National Traffic injury Rehabilitation Hospital, Yangpyeong, Gyeonggi-do, Republic of Korea; 3 Department of Neuropsychiatry, Seoul National University Hospital, Seoul, Republic of Korea; 4 Department of Psychiatry, College of Medicine, Seoul National University, Seoul, Republic of Korea; 5 School of Digital Humanities and Computational Social Sciences, Korea Advanced Institute of Science and Technology, N4 Bldg, Room 1314, 291 Daehak-ro, Yuseong-gu, Daejeon, 34141, Republic of Korea, 82 010-9797-0807; 6 School of Liberal Arts, Ulsan National Institute of Science and Technology, Ulsan, Republic of Korea; 7 Jeonbuk National University Hospital, Jeollabuk-do, Republic of Korea; 8 Department of Psychiatry, Sungkyunkwan University School of Medicine, Kangbuk Samsung Hospital, Seoul, Republic of Korea; 9 Mind Care and Growth Center, Korea Advanced Institute of Science and Technology, Daejeon, Republic of Korea; 10 Department of Psychiatry, College of Medicine, Korea University, Seoul, Republic of Korea; 11 Department of Biomedical Informatics, College of Medicine, Korea University, Seoul, Republic of Korea; 12 Department of Medical Education, College of Medicine, Korea University, Seoul, Republic of Korea

**Keywords:** mental health artificial intelligence, generative artificial intelligence, psychiatrists, qualitative research, thematic analysis, horizon scanning

## Abstract

**Background:**

Generative artificial intelligence (GenAI) has increasingly entered psychiatric practice through patient-facing chatbots, self-help tools, and clinician-facing workflow support. Although prior research has examined clinicians’ attitudes, readiness, and anticipated use cases, less is known about how frontline encounters with GenAI shape psychiatrists’ interpretations and implementation priorities. Health care foresight also remains methodologically underdeveloped and has focused mainly on external signals, overlooking clinically consequential signals emerging from everyday practice. This gap is especially important in psychiatry, where GenAI-related benefits and harms may depend on patient vulnerability, crisis sensitivity, and the therapeutic relationship.

**Objective:**

This study aims to qualitatively examine how South Korean psychiatrists described clinical experiences with GenAI, how they interpreted its roles and limits in psychiatric care, and what implementation priorities they emphasized. Selected concepts from horizon scanning informed the organization of the analysis by orienting attention to practice-based signals, interpretive patterns, and implementation priorities.

**Methods:**

In this qualitative descriptive study, directed content analysis and codebook-based thematic synthesis were used to analyze responses to 3 open-ended survey questions administered to members of the Korean Neuropsychiatric Association. Invitations were distributed through the association’s official email system from October 27 to December 26, 2025. The qualitative analysis included respondents who provided an interpretable response to at least 1 item. The questions addressed (1) GenAI-related clinical experiences, (2) perceived advantages and limitations of GenAI relative to human therapists, and (3) priorities for the safe introduction of GenAI into mental health care. An exploratory participant-level cross-question thematic alignment analysis was also conducted to examine recurring adjacent-item pairings across the experience-interpretation-priority sequence.

**Results:**

Of 408 total survey respondents, 311 respondents provided a meaningful response to at least 1 open-ended item. Psychiatrists described GenAI as a clinically ambivalent technology whose implications depended on context, intensity of use, and patient vulnerability. Practice-based signals clustered around patient-led use, clinician-led use, GenAI as a relational object, and GenAI-mediated changes in the patient-clinician interface, with high-risk and destabilizing scenarios cutting across these themes. Respondents viewed GenAI as potentially useful as an adjunct, but also as relationally limited and unacceptable as a replacement for human therapists. Implementation priorities centered on governance, crisis and vulnerability safeguards, technical reliability and clinical validation, and education, supervision, and structural readiness. Cross-question analysis suggested recurrent alignments between frontline signals, a view of GenAI as standardized and tireless but relationally thin, and governance- and validation-oriented implementation priorities.

**Conclusions:**

In this qualitative descriptive study, GenAI emerged in psychiatric practice as an access tool, a workflow aid, and, at times, a competing interpretive reference point in clinical encounters. The key implementation challenge is therefore not whether psychiatry will encounter GenAI, but how its use should be bounded, supervised, and governed in light of patient vulnerability, psychiatric risk, and the relational demands of care.

## Introduction

In this digital era, generative artificial intelligence (GenAI) is becoming embedded in everyday life and psychiatric systems, and is entering psychiatric practice through patient-facing conversational tools, self-help applications, and clinician-facing workflow support [1-3]. Psychiatrists are therefore likely to encounter GenAI not as a distant technological prospect, but as a technology already shaping how patients interpret distress, seek help, and present to the clinic. Recent studies suggest that clinicians are not uniformly resistant to artificial intelligence (AI); rather, they tend to view it as practically useful for documentation tasks, information processing, and other efficiency-oriented functions, while remaining far more cautious about its use in psychotherapy-like or relationship-centered care [4-9].

Given the complexity surrounding patient vulnerability, reality testing, crisis sensitivity, and the therapeutic relationship itself in psychiatry, the relevance of GenAI in psychiatric practice cannot be judged solely in terms of usability, convenience, or technical fluency. Recent work has similarly emphasized that conversational AI in health-related settings should be evaluated not only for performance or convenience, but also for risk-sensitive reasoning and oversight requirements [10]. Moreover, emerging literature has raised concerns that GenAI chatbots may reinforce delusion-like beliefs, provide excessive validation of distorted interpretations, or respond inadequately to suicide-related and other high-risk scenarios [11-17].

Additional work has suggested that prolonged or emotionally salient chatbot use may also be associated with dependence-like patterns, social withdrawal, or psychosocial deterioration in vulnerable users [13,14]. Taken together, these findings suggest that the clinical implications of GenAI in psychiatry are not reducible to generic benefits of access or conversational convenience. Rather, they depend on whether these tools are introduced into situations requiring contextual judgment, relational attunement, and the capacity to recognize and contain risk [2,15,16].

Much of the current literature has focused on clinicians’ attitudes, readiness, trust, adoption intentions, and anticipated applications of GenAI in mental health care [4-9]. Less insight is available into how concrete frontline encounters with GenAI are interpreted by psychiatrists in practice, how different types of experiences shape professional judgments about GenAI’s proper role, and how such judgments translate into priorities for supervision, governance, and implementation. Related qualitative work further suggests that physicians expect GenAI to affect the therapeutic relationship in both supportive and disruptive ways, including through altered trust, transparency, and shared decision-making [18]. Yet the field still lacks an approach that systematically links reported clinical signals with broader interpretive patterns and future-oriented implementation demands.

At a methodological level, similar concerns have been raised regarding health care foresight. In a recent scoping review, Meskó et al [9] found that only a limited subset of futures and foresight methods has been used in medicine and health care, and that their theoretical and methodological foundations remain insufficiently developed. Traditional horizon scanning, in particular, has been criticized for being less sensitive to weak signals first emerging within everyday clinical encounters and for generally focusing on external sources (eg, publications, patents, policies).

This limitation may be especially important in psychiatry, where early signs of benefit, boundary tension, or harm may first emerge in everyday interactions between patients, clinicians, and digital tools rather than in formal evidence hierarchies. In this sense, psychiatric foresight may need to begin with practice-based signals, not external innovation indicators alone [19].

South Korea provides a particularly relevant context for examining these questions. The country combines high digital uptake with persistent gaps in treatment utilization for mental disorders [20,21]. Help-seeking is shaped not only by stigma, but also by structural concerns related to psychiatric records, employment disadvantage, and other forms of institutional discrimination [22]. In this context, anonymous, immediate, and low-threshold AI-based support may be especially attractive to individuals reluctant to seek formal psychiatric care. At the same time, these conditions may increase the likelihood of GenAI being used without adequate clinical oversight, intensifying concerns about safety, scope, and accountability. Recent global guidance has emphasized that large multimodal AI systems in health require stronger governance, transparency, risk management, and human accountability [22], yet how these principles should be translated into the realities of psychiatric practice remains underdeveloped. This concern is also consistent with recent AI governance scholarship arguing that responsible deployment requires deeper value-oriented oversight structures beyond external constraints alone [23].

To address these gaps, this study draws on horizon scanning concepts to organize a qualitative analysis of open-ended responses from psychiatrists in South Korea. The aim was to map practice-based signals arising in clinical encounters, identify broader interpretive patterns regarding the roles and limits of GenAI in psychiatric care, and characterize future-oriented implementation priorities. Rather than examining attitudes toward GenAI in the abstract, we sought to identify clinically salient frontline signals, characterize how psychiatrists interpret the roles and limits of GenAI in mental health care, and examine how these patterns relate to future implementation. We also examined how themes aligned across the connected analytic domains of experience, interpretation, and implementation priority. By linking experience, interpretation, and implementation priorities within a single analytic structure, this study aims to contribute empirically by clarifying how psychiatrists are already encountering GenAI in practice, and methodologically by illustrating how horizon scanning concepts can be adapted for practice-based signal mapping in psychiatry [9].

Building on these aims, this study contributes in several ways by clarifying key patterns in how GenAI is interpreted and implemented in psychiatric practice. Specifically, it (1) shows that clinicians’ interpretations of GenAI are context-dependent and shaped by psychiatric vulnerability, (2) provides a structured account of the implementation gap through an access–protection tension, and (3) demonstrates a practice-based approach to horizon scanning using clinician-reported experiential data.

## Methods

### Study Design

This qualitative descriptive study analyzed open-ended survey responses using directed content analysis and codebook-based thematic synthesis. Selected concepts from horizon scanning informed the organization of the analysis across 3 linked domains: practice-based signals, interpretive patterns, and implementation priorities [19]. Reporting was guided by the Standards for Reporting Qualitative Research [24], and the description of theme development and analytic procedures was written with attention to recent guidance on transparent thematic analysis reporting [25].

### Setting and Participants

From October 27 to December 26, 2025, invitations to participate were distributed 5 times through the official email system of the Korean Neuropsychiatric Association to all registered members. A total of 408 participants, including 326 board-certified psychiatrists and 82 residents, were included in the overall survey sample.

For the qualitative component, respondents were eligible if they provided an interpretable response to at least 1 of the 3 open-ended questions. Because each item was analyzed separately, the analytic denominator varied by question. Respondents who did not answer all 3 questions were still retained in the qualitative dataset if they contributed interpretable text to at least 1 item, because a single response could still provide meaningful insight relevant to the corresponding analytic domain.

Exclusion was applied at the item level rather than the respondent level. Brief responses were not excluded solely because of length; single-word or short responses were retained if they were semantically interpretable and directly addressed the question. Responses were excluded only when both coders judged them to be noninterpretable, off-topic, or nonsubstantive, with disagreements resolved through discussion.

### Open-Ended Survey Questions

The present analysis was restricted to the following 3 open-ended survey questions. Below are English translations of the original Korean survey questions:

- Q1: “Please freely share any memorable event or experience related to generative AI technologies in clinical practice, such as chatbots or diagnostic support tools.”
- Q2: “Please describe your views on the unique advantages and limitations of generative AI in mental health care compared with human therapists.”
- Q3: “Please list up to 3 tasks that you believe are most urgent for the safe and effective introduction of generative AI technologies into mental health care.”

Although each question was designed to stand on its own, the item sequence was intentionally structured to align with the study’s analytic framework across experience, interpretation, and implementation priority. These corresponded to the study’s 3 analytic domains: practice-based signals, interpretive patterns, and implementation priorities.

Although each question was designed to stand on its own, the item sequence was intentionally structured to align with the study’s 3 analytic domains: practice-based signals, interpretive patterns, and implementation priorities.

### Use of Selected Horizon Scanning Concepts for Practice-Based Signal Mapping

This study drew on selected concepts from horizon scanning as an organizing heuristic. Two elements were adapted: first, the notion of practice-based signals, namely clinically consequential early indicators emerging within routine care before they appear in formal evidence hierarchies; and second, a 3-layer structure linking practice-based signals, interpretive patterns, and implementation priorities. Unlike conventional horizon scanning, which relies on external data sources such as publications, patents, and policy documents, this study used clinicians’ open-ended survey responses as the signal source and qualitative thematic analysis as the analytic method.

At the first layer, Q1 responses were examined as practice-based signals arising from clinical encounters, drawing conceptually on selected horizon scanning concepts [19]. At the second layer, Q2 responses were synthesized thematically to identify interpretive patterns regarding the perceived roles, limits, strengths, and boundaries of GenAI in mental health care relative to human therapists [26]. At the third layer, Q3 responses were synthesized to identify implementation priorities for the safer and more effective introduction of GenAI into mental health care [25]. Without imposing an a priori coding framework, responses were inductively coded and synthesized into broader implementation-priority themes reflecting clinicians’ future-oriented concerns.

Integration across the 3 layers was conducted through cross-question analysis at the participant level. By linking each respondent’s codes across Q1, Q2, and Q3, we explored recurring within-respondent alignments across the connected analytic domains of experience, interpretation, and implementation priority, with particular attention to alignments between adjacent domains.

In this study, horizon scanning concepts were used only to organize analytic attention, whereas coding, theme development, and synthesis remained grounded in qualitative content analysis and thematic synthesis.

### Research Team and Reflexivity

The qualitative analysis was conducted by a multidisciplinary team with expertise in psychiatry, digital health, clinical psychology, nursing, biomedical engineering, and technology-enabled health research. MK, a doctoral student researcher with prior mixed methods experience in emerging digital mental health technologies, coordinated the analytic process. During the initial familiarization and open-coding phase, MK worked with YL and HK to review responses and generate preliminary data-near codes. MJ and YL, both of whom had prior supervised experience in qualitative analysis within digital health research, then conducted the main independent coding under MK’s ongoing oversight.

Because team members differed in disciplinary background and prior qualitative experience, the analysis emphasized explicit code definitions, iterative discussion of coding boundaries, and repeated review of discrepant cases to strengthen consistency and reflexive awareness. Psychiatrist investigators (DJ, YA, and CHC) contributed clinical judgment regarding the plausibility and psychiatric significance of reported events, whereas nonphysician researchers focused on coding transparency, category boundaries, and interpretive consistency across responses. Reflexive discussions explicitly considered assumptions related to technological optimism, patient safety, psychiatric vulnerability, and professional boundary concerns throughout coding and theme development [27,28].

### Codebook Development

Codebook development proceeded in stages using a combined inductive-deductive approach derived from directed qualitative content analysis [29]. First, all responses to each question were reviewed through open coding to generate an initial pool of data-near codes. This stage was conducted under the coordination of MK, with YL and HK contributing to preliminary code generation for each item.

Second, MK, MJ, and YL organized and refined these preliminary codes into separate item-specific codebooks. This refinement process was informed both by the data and by sensitizing concepts drawn from the literature, including clinical usefulness of GenAI, categories of risk, therapeutic relationship, and concepts from horizon scanning and technology adoption. Through this combined inductive-deductive process, separate codebooks were established for each question, each containing code labels, operational definitions, inclusion criteria, exclusion criteria, and illustrative quotations.

The preliminary codebooks were piloted on a subset of responses from each question and iteratively revised before full coding. During piloting, the team specifically reviewed whether meaning units were too broad or too narrow, whether adjacent codes overlapped conceptually, and whether brief responses could be coded consistently under the proposed definitions. Where ambiguities were identified, code labels, definitions, and decision rules were revised accordingly. This iterative process was informed by qualitative content analysis literature emphasizing that abstraction and interpretation are inseparable from the processes of condensing, coding, and forming categories [27,28]. Once consensus had been reached on code definitions and coding rules, the codebooks were fixed for the main coding phase, with only minor clarifications permitted thereafter.

A detailed audit trail of codebook refinement, discrepant-code review, and code-level intercoder agreement across coding rounds is provided in Multimedia Appendix 1.

### Coding Procedure

Coding was conducted independently by MJ and YL. The analytic unit was the individual response to each open-ended question, with coding decisions made at the level of meaning units, allowing multiple codes to be assigned within a response when distinct ideas were identified.

For the first question, coders assigned all applicable substantive codes and also coded experiential proximity. For the second question, coders initially coded statements describing advantages and limitations separately. Rather than binary categories, codes were integrated to reflect that GenAI features often present simultaneous advantages and limitations depending on the clinical context. This coexistence was treated as central to interpretation rather than as a coding problem. For the third question, codes were assigned inductively to the implementation issues and priorities raised by respondents, without applying predefined categories of challenge type or urgency.

After an initial round of independent coding, coder consistency was reviewed at the response level using Cohen’s kappa. This step was used pragmatically to identify ambiguous code boundaries and refine code definitions during codebook development, rather than to imply a purely positivist model of qualitative rigor [30,31]. For codes with Cohen’s kappa values below 0.70, MJ and YL reviewed discrepant cases with MK to identify the source of disagreement. Relevant codebook definitions and decision rules were then refined, and affected responses were recoded before final agreement statistics were calculated. Final code assignments were determined through consensus discussion rather than by excluding low-agreement codes from analysis. This iterative refinement process was designed to improve coding consistency while preserving analytically important distinctions in the data [27-29].

Following completion of the coding phase, YA participated in theme development and interpretive synthesis as a psychiatrist investigator. At this stage, YA contributed clinical judgment regarding the psychiatric significance of code patterns, the plausibility of reported clinical scenarios, and the linkage between coded response patterns and implications for psychiatric practice. Detailed information on changes in intercoder agreement and codebook refinement across coding rounds is reported in Multimedia Appendix 1.

### Thematic Synthesis

Following item-level coding, codes were grouped into subthemes and broader themes through team-based thematic synthesis. Braun and Clarke’s broad phases of familiarization, coding, theme development, review, and naming informed the organization of this process, while the analysis remained grounded in a qualitative descriptive, codebook-based approach [26]. Within each question, codes were grouped into subthemes according to shared clinical meaning, interpretive orientation, or implementation relevance, and these subthemes were then synthesized into broader themes.

For the first question, the thematic structure was interpreted in relation to signal content and experiential proximity. For the second question, themes were developed to capture broader interpretive patterns in respondents’ views of GenAI in mental health care, including tensions, overlaps, and boundary judgments within the same thematic domain. Accordingly, potentially positive and negative aspects of a given feature were retained together when they formed part of a single coherent theme. For the third question, themes were developed inductively to represent clinicians’ perceived implementation priorities and governance.

Theme development was conducted through joint review and interpretive synthesis by the research team after completion of coding. In this phase, psychiatrist and nonpsychiatrist investigators contributed different but complementary perspectives. More precisely, the former emphasized clinical plausibility, contextual meaning, and implications for psychiatric care, whereas the latter focused on thematic coherence, transparency of analytic transitions, and fidelity to the wording of the responses. Recent guidance on transparent thematic analysis reporting informed the description of these analytic steps in the manuscript [25].

### Exploratory Cross-Question Thematic Alignment Analysis

Cross-question analysis was conducted using a participant-level coding matrix. For each respondent, the matrix linked assigned codes across the 3 open-ended questions. Because quantified co-occurrence is not typically presented as a primary output of thematic analysis, the coding matrix was used to examine cross-item theme pairings as supplementary descriptive indicators. Specifically, we examined how particular alignments recurred across respondents, whether some pairings showed relatively dense overlap after accounting for base prevalence, and whether alignment patterns differed across adjacent items. This rationale was informed by prior work suggesting that thematic co-occurrence can help clarify not only whether themes overlap, but also how their relationships are patterned, asymmetric, and interpretively meaningful [32], as well as by qualitative-quantitative approaches using co-occurrence matrices or coefficients to support exploratory interpretation of coded qualitative data [33,34]. Accordingly, for each adjacent-item theme pair (Q1-Q2 and Q2-Q3), we calculated raw co-occurrence counts, directional conditional proportions, and a prevalence-adjusted overlap index based on the Jaccard (c-coefficient) formula: nAB/(nA+nB−nAB). These indices were interpreted descriptively rather than inferentially. They were used to support narrative findings about recurrent and directionally patterned within-respondent thematic alignments, especially where relatively high-overlap pairings appeared to reinforce an existing concern, add a complementary explanatory layer, or connect frontline experience with future implementation priorities [34]. Consistent with thematic analysis, frequency was not treated as a proxy for thematic importance [35].

### Rigor and Trustworthiness

Rigor was addressed using the criteria proposed by Lincoln and Guba [36]. Credibility was strengthened through independent double coding, iterative consensus discussions, involvement of multidisciplinary investigators, and the inclusion of illustrative quotations. Transferability was supported by a detailed description of participant characteristics and attention to the Korean clinical and regulatory context. Dependability was enhanced through explicit codebook development, documented refinement of code definitions and decision rules, and maintenance of an audit trail for coding discrepancies and revisions. Confirmability was addressed by distinguishing clearly between data-grounded coding decisions, theory-informed sensitizing concepts, and later interpretive synthesis.

Because the study concerned an emerging technology associated with both enthusiasm and alarm in public discourse, reflexive discussion formed an explicit part of the analytic process. Team members discussed potential preconceptions regarding technological optimism, patient safety, psychiatric vulnerability, and professional boundary concerns before and during analysis. These discussions informed codebook refinement and theme review, but were not used to override the data. In addition, the iterative review of discrepant codes and recoding of affected responses helped make analytic decisions more transparent and less dependent on any single coder’s assumptions [24,25,27,28].

### Ethical Considerations

This study was approved by the Seoul National University Hospital Institutional Review Board (approval number SNUH IRB 2025-10-001). All responses were collected and analyzed anonymously, and electronic informed consent was obtained from all participants before survey initiation. Participants who completed the survey received a small incentive in the form of a 10,000 (USD $6.82) KRW Starbucks mobile gift voucher.

## Results

### Overview of Qualitative Responses

Of the 408 survey participants, 311 provided a meaningful response to at least 1 of the 3 open-ended questions after application of the predefined item-level exclusion criteria and were included in the qualitative analytic sample. Meaningful responses were available for 218 participants in Q1, 232 in Q2, and 220 in Q3. Demographic and professional characteristics of the qualitative sample are presented in Table 1.

**Table 1.** Demographic and professional characteristics of the qualitative analytic sample.

Variable	Total^a^ (n=311)	Q1 (n=218)	Q2 (n=232)	Q3 (n=220)	Overlap^b^ (n=129)
Age (years), mean (SD)	42.81 (9.89)	41.9 (9.23)	43.76 (9.80)	43.89 (10.04)	43.32 (9.14)
Clinical experience (years), mean (SD)	14.7 (9.90)	13.91 (9.16)	15.39 (9.84)	15.66 (10.14)	15.03 (9.08)
Sex, n (%)					
Male	197 (63.34)	135 (61.93)	143 (61.64)	136 (61.82)	76 (58.91)
Female	114 (36.66)	83 (38.07)	89 (38.36)	84 (38.18)	53 (41.09)
Professional status, n (%)					
Specialist	256 (82.32)	178 (81.65)	198 (85.34)	186 (84.55)	110 (85.27)
Resident	55 (17.68)	40 (18.35)	34 (14.66)	34 (15.45)	19 (14.73)
Workplace setting, n (%)					
Tertiary hospital	101 (32.48)	66 (30.28)	67 (28.88)	66 (30.00)	31 (24.03)
General hospital	37 (11.90)	27 (12.39)	28 (12.07)	28 (12.73)	17 (13.18)
Hospital	6 (1.93)	5 (2.29)	2 (0.86)	2 (0.91)	1 (0.78)
Psychiatric hospital	42 (13.50)	28 (12.84)	33 (14.22)	31 (14.09)	18 (13.95)
Clinic	120 (38.59)	90 (41.28)	99 (42.67)	90 (40.91)	62 (48.06)
Other	5 (1.61)	2 (0.92)	3 (1.29)	3 (1.36)	0 (0.00)

a Total refers to the set of participants who provided at least one meaningful response to Q1, Q2, or Q3.

b Overlap indicates respondents who provided meaningful responses to all 3 open-ended questions. In addition, pairwise overlaps (including respondents who also answered all three questions) were observed between Q1 and Q2 (n=144), Q2 and Q3 (n=210), and Q1 and Q3 (n=134).

**Figure 1.**
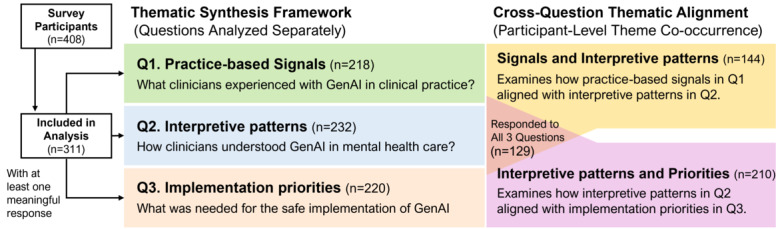
Structure of the qualitative results and cross-question thematic alignment. GenAI: generative artificial intelligence.

Figure 1 provides a brief overview of the structure of the qualitative findings, showing the number of respondents contributing to each question-specific section and to the cross-question comparisons. The Results first present themes identified independently for Q1, Q2, and Q3, addressing practice-based signals, interpretive patterns, and implementation priorities, respectively, and then turn to an exploratory analysis of how themes aligned across adjacent question pairs at the participant level, particularly between Q1 and Q2 and between Q2 and Q3. Participant quotations were translated from Korean into English and lightly edited for readability while preserving the original meaning and tone. Quotations are identified by participant number, indicated as P followed by the respondent number (eg, P217).

### Practice-Based Signals Emerging From Clinical Encounters With GenAI (Q1)

#### Overview

Responses to Q1 suggested that psychiatrists’ experiences with GenAI were organized less by a simple positive-versus-negative distinction than by how GenAI entered and functioned within care. Reported experiences clustered around four themes, with high-risk and destabilizing scenarios cutting across them. Across these themes, bounded or lower-intensity use tended to be described as facilitative, whereas higher-intensity use, overreliance, or transfer of interpretive authority to AI tended to be associated with symptom aggravation, treatment disruption, and safety concerns.

#### Patient-Led Signals: Self-Help, Triage, and Early Gateway Use

The first set of signals involved patient-initiated use of GenAI as a self-help, reflective, triage, or information-checking tool. In these responses, GenAI was generally described not as a substitute for treatment but as a low-threshold aid that sometimes helped patients organize emotions, consider whether professional help might be needed, or bring questions about AI use back into clinical care.

*I saw a patient who did not use ChatGPT simply for comfort, but wrote out their situation and the emotions they were feeling at the time, and then asked it not to comfort them but to help organize them. Through this, the patient was able to verbalize emotions that had been difficult to process, and I confirmed that the symptoms improved.*

[P217]

Another respondent described AI as prompting treatment entry:

*The patient came after being advised during an interview with AI to seek care*

[P74]

These responses suggested that psychiatrists were already encountering AI as a preliminary reflective or gateway tool before formal psychiatric contact. However, when patient-led use became more intense or occurred in higher-risk situations, respondents typically described deterioration or safety concerns rather than benefit. Reported experiences included reinforcement of delusion-like beliefs, worsening of nonpsychotic symptoms, exposure to inappropriate or unverified AI services, and suicide- or self-harm-related risk.

*A patient once asked ChatGPT and, after being told the medication was not a high dose, took an overdose for self-harm.*

[P20]

At the same time, some responses indicated that AI could also facilitate help-seeking in the opposite direction, such as when a patient with suicidal ideation received a crisis hotline number during chatbot use and was subsequently linked to services (P157). Overall, patient-led use appeared acceptable mainly when GenAI remained a preliminary aid rather than an unsupervised guide during vulnerable states.

#### Clinician-Led Signals: Workflow Support, Clinical Reasoning, and Bounded Experimentation

A second set of signals came from clinicians’ own use of AI, which was described mainly in practical and workflow-oriented terms. Respondents reported using AI to summarize records, prepare educational materials, compare medications, and obtain rapid information or informal support, particularly for tasks experienced as structured, repetitive, or information dense.

*When I used a chatbot to prepare educational materials or video materials for patients or caregivers, it helped me prepare them quickly with less effort. … Using a chatbot to create the basic outline and then having an expert review it made efficient work possible*

[P230]

Another respondent noted that many physicians had likely already used GenAI informally to check drug interactions when the answer did not immediately come to mind (P224). These responses suggested that clinicians regarded GenAI as more appropriate for supportive and reviewable tasks than for independent psychiatric decision-making. Some emphasized hallucinated outputs, factual inaccuracy, and contextually inappropriate recommendations. One psychiatrist who had used paid general-purpose ChatGPT for literature and medication-related queries reported that it was useful in outpatient practice, but that medically inaccurate statements and even serious errors were “often noticeable,” concluding that it was still too early to introduce such systems to the public for counseling, diagnosis, or treatment without expert review (P135). In short, clinicians accepted GenAI mainly for structured, reviewable tasks under human oversight.

#### GenAI as a Relational Object: Attachment, Substitution, and Social Functioning

A third pattern concerned GenAI not only as a tool but also as a quasi-relational object. Respondents described cases in which GenAI appeared to provide companionship, a psychologically safer space for disclosure, or support for self-expression and behavior change, particularly among socially isolated or inhibited patients.

*A socially isolated patient whose sense of isolation was alleviated through chatbot use*

[P24]

Another psychiatrist described a patient who had rarely disclosed inner thoughts to anyone, but who, after using a chatbot, became more able to express thoughts and feelings during treatment and even reported trying new interpersonal behaviors with the chatbot’s help (P200). GenAI sometimes functioned as a relational scaffold rather than only as a technical aid, but these same features became concerning when reliance deepened, or symptom worsening coincided with intensified use.

*In patients, the amount of conversation with ChatGPT increased whenever they relapsed. I thought that by monitoring the interaction itself, it might be possible to predict relapse*

[P22]

Other respondents described patients saying that the chatbot understood them better than family members, or spending most of the day in conversation with it. In these cases, GenAI appeared to move beyond supportive aid and occupy a more central emotional or behavioral role, raising concerns about overdependence, social withdrawal, and reduced offline coping or relational capacity.

#### GenAI-Mediated Changes in the Patient-Clinician Interface

A fourth theme concerned ways in which GenAI was beginning to reshape the consultation itself. Respondents described patients arriving with AI-generated summaries, discussing prior chatbot interactions during visits, or using GenAI output to organize what they wanted to say to the psychiatrist. In lower-intensity encounters, AI sometimes facilitated communication and more efficient use of limited clinic time.

*There was a patient who routinely discussed concerns with AI and, in the outpatient clinic, summarized those conversations and asked the treating physician’s opinion. The patient was highly intelligent, and the conversations with AI dealt with clinically important topics, so I found it impressive that this seemed to use the limited clinic time efficiently*

[P135]

At the same time, respondents also described more disruptive interface experiences in which GenAI became a comparator or competing interpretive reference point. Patients were reported to cross-check clinicians’ advice with chatbot output, request medications based on GenAI recommendations, or compare the therapist’s responses with those of a chatbot. In some cases, this reinforced trust when GenAI and the clinician converged, but in others it undermined diagnostic authority, treatment adherence, or the therapeutic relationship itself. Overall, AI entered the consultation not only as information, but also as a competing reference point that reshaped verification, authority, and trust.

*A patient came to me angry after telling ChatGPT their symptoms and receiving an answer about the diagnosis, saying that their attending psychiatrist had given a different diagnosis and that they could no longer trust the attending physician. In the end, the attending psychiatrist’s diagnosis was correct, and the problem had arisen because the patient mentioned only biased symptoms, that is, only those they themselves acknowledged*

[P53]

### Interpretive Themes Regarding GenAI in Mental Health Care (Q2)

#### Overview

Responses to Q2, which focused on GenAI in mental health care, did not separate neatly into advantages and limitations. Instead, the same properties, such as availability, nonjudgment, and standardized responding, were often described as beneficial in one clinical context and problematic in another. Four integrated themes were identified. Respondents’ judgments appeared to depend less on whether a feature was inherently advantageous or problematic than on whether it could be appropriately bounded, contextualized, and assigned a clinically suitable role.

#### GenAI as a Low-Threshold and Always-Available Point of Contact

Many respondents viewed GenAI as uniquely accessible relative to human therapists. They emphasized its availability across time and place, immediate responsiveness, low cost, and reduced stigma through anonymity. These features were seen as particularly relevant in situations where conventional mental health care remained difficult to access or where patients hesitated to disclose symptoms to another person. In this sense, GenAI was interpreted as a low-threshold conversational entry that reduces barriers to contact.

*AI chatbots have the advantage of immediacy and accessibility… Therefore, they should be understood not as therapeutic replacements, but as entry points for those who cannot access treatment and as tools that support human care.*

[P33]

#### Standardized and Tireless, but Relationally Thin

Respondents also described GenAI as offering consistency, neutrality, and freedom from fatigue, countertransference, or mood-dependent variability. Some regarded these qualities as strengths, particularly for structured psychoeducation, repetitive support, or protocol-based interventions.

*The strengths of AI chatbots are that they are standardized, evidence-based, and less prone to error. Their limitations are the lack of individualized responses that respect each person’s uniqueness and the limited ability to convey empathy.*

[P15]

However, these perceived strengths were frequently contrasted with the limitations of GenAI as a relational agent. Respondents emphasized that GenAI could not adequately perceive nonverbal cues, grasp emotional nuance in the same way as a human therapist, or participate in the intersubjective processes through which therapeutic change often occurs. These strengths were simultaneously interpreted as signs of relational thinness and therapeutic incompleteness. At the same time, several responses implied that although some limitations might be improved technically, others were more fundamentally tied to the absence of human intersubjectivity, warmth, and embodied perception in psychiatric care.

#### Nonjudgmental Acceptance as Both Comfort and Clinical Hazard

A recurrent tension concerned GenAI’s nonjudgmental and continuously accepting stance. Respondents viewed this nonjudgmental stance as double-edged: it could facilitate disclosure, but it could also become clinically hazardous.

*Its advantages are that it is always available and allows disclosure of shameful content. Its risks are that users may hear only what they want to hear, while delusional thinking may be reinforced and social isolation may increase.*

[P229]

They worried that GenAI might provide users with the answers they wanted to hear, fail to deliver necessary therapeutic challenge, or respond in an overly validating manner to distorted or dangerous beliefs. In this sense, respondents suggested that the problem was not support itself, but support that was insufficiently modulated by doubt, limit-setting, or reality testing. This concern became especially pronounced in relation to psychosis, delusion-like ideation, and suicide or self-harm risk, where respondents emphasized that apparently supportive responses could worsen, rather than contain, vulnerability.

#### Useful as an Adjunct, Not Acceptable as a Replacement

Across responses, acceptance of the GenAI chatbot was generally conditional and adjunctive rather than substitutive. Respondents were more comfortable assigning GenAI a supportive role than accepting it as an independent therapeutic agent. This bounded stance was closely tied to perceived limitations in contextual understanding, individualized formulation, accountability, and supervision. Many respondents suggested that once GenAI moved beyond support functions and began to occupy the position of therapist, diagnostic authority, or crisis responder, its limitations became substantially more concerning.

*In conclusion, AI chatbots are most effective when used as a complement to, rather than a replacement for, human therapists.*

[P388]

### Implementation Priorities for Safer Psychiatric Use of GenAI (Q3)

#### Overview

Q3 translated these interpretive tensions into future-oriented implementation priorities for action. Rather than primarily calling for wider deployment, respondents emphasized the conditions under which GenAI could be introduced safely, legitimately, and responsibly in psychiatric settings. Four strategic priority themes were identified: governance and accountability, safety protections for high-risk groups, technical and evidentiary reliability, and educational or structural readiness. Taken together, these priorities were framed less as a roadmap for expansion than as preconditions for legitimate and safer use in psychiatric settings.

#### Governance and Accountability as Prerequisites for Adoption

Governance and accountability emerged as foundational prerequisites for adoption. Respondents frequently called for ethical standards, practical guidelines, legal frameworks, and clearer allocation of responsibility in the event of harm.

*There should be a basic code of ethics for AI technology, along with practical guidelines and a supervisory body to ensure that it is used appropriately.*

[P32]

These concerns extended beyond abstract principles to the operational question of who should be accountable when GenAI contributes to inaccurate advice, delayed intervention, privacy breaches, or other adverse outcomes. Many responses suggested that, in the absence of professional and regulatory scaffolding, broader implementation would be premature. Some responses also emphasized privacy and data protection, particularly given the sensitivity of psychiatric information and concerns about clinical or counseling content being entered into external GenAI systems without clear safeguards.

#### Safety Infrastructures for Crisis Situations and Vulnerable Populations

Safety infrastructures for high-risk scenarios and vulnerable groups were identified as essential implementation requirements. Respondents emphasized the need for systems capable of identifying suicidality, self-harm risk, or other emergencies and linking users to timely intervention.

*Institutional safeguards are needed for patient safety, such as automatic reporting to relevant agencies or professionals when warning signs related to self-harm or harm to others are identified.*

[P135]

They also highlighted the need for safeguards specific to psychosis and other conditions involving impaired reality testing, where AI’s tendency toward excessive agreement or uncritical responding was seen as particularly dangerous. These responses suggested that clinicians did not view safety as a generic design feature, but as something that must be tailored to psychiatric risk profiles and clearly defined contraindications.

#### Technical Reliability and Clinical Validation Before Scale-Up

Respondents also emphasized that implementation should depend on technical and evidentiary maturity rather than novelty alone. Priorities in this theme included improving information accuracy, reducing hallucinations, developing mental health-specific systems, and validating safety and effectiveness through clinical research.

*There needs to be a way to address hallucinations so that the system responds only on the basis of evidence-based materials.*

[P82]

*It should undergo efficacy and safety testing based on validated content.*

[P124]

Some responses additionally pointed to the need for standardized therapeutic content or protocols so that AI-supported mental health care would not depend solely on the behavior of general-purpose models. Technical performance and clinical legitimacy were treated as inseparable requirements for adoption.

#### Education, Supervision, and Structural Support for Responsible Use

Education, supervision, and structural readiness were also identified as necessary conditions for responsible use. Respondents called for clinician education about the capabilities and limits of GenAI, better public understanding of what mental health GenAI tools can and cannot do, and mechanisms for expert monitoring or audit of AI-assisted care.

*Both patients and physicians need to use AI with an awareness of its limitations, so related education and oversight at the level of academic societies or government are necessary.*

[P177]

Some respondents also referred to the practical infrastructure needed for implementation, including reimbursement, institutional support, and integration into existing systems. Together, these responses suggested that responsible use depends not only on technical design or regulation, but also on user competence, professional oversight, sustainable clinical integration, and clearer boundaries of indication and scope.

### Within-Respondent Patterns Across Experience, Interpretation, and Priority

Mapping themes across adjacent items revealed several recurrent alignments between clinical experience, interpretation, and implementation priority. Full pairwise alignment matrices, including raw co-occurrence counts, directional conditional proportions, and Jaccard coefficients, are presented in Multimedia Appendix 2.

Across Q1-Q2, several practice-based signals aligned most strongly with the interpretive theme of standardized and tireless, but relationally thin AI, with the highest Jaccard overlap observed for patient-led signals (Jaccard=0.190), AI-mediated changes in the patient-clinician interface (Jaccard=0.160), and AI as a relational object (Jaccard=0.149; Table S1 in Multimedia Appendix 2). Table S1 in Multimedia Appendix 2 further suggests that AI as a relational object also formed a relatively specific pairing with nonjudgmental acceptance as both comfort and clinical hazard (Jaccard=0.164), indicating that relationally salient experiences were interpreted not only in terms of limited depth or standardization, but also in terms of the chatbot’s continuously accepting stance.

Across Q2-Q3, the interpretive theme of standardized and tireless, but relationally thin AI showed the clearest overlap with governance and accountability as prerequisites for adoption (Jaccard=0.447), technical reliability and clinical validation before scale-up (Jaccard=0.360), and education, supervision, and structural support for responsible use (Jaccard=0.291; Table S2 in Multimedia Appendix 2). These pairings indicate that concerns about the relational and contextual limits of AI were closely linked to future-oriented demands for oversight, validation, and bounded implementation.

**Figure 2.**
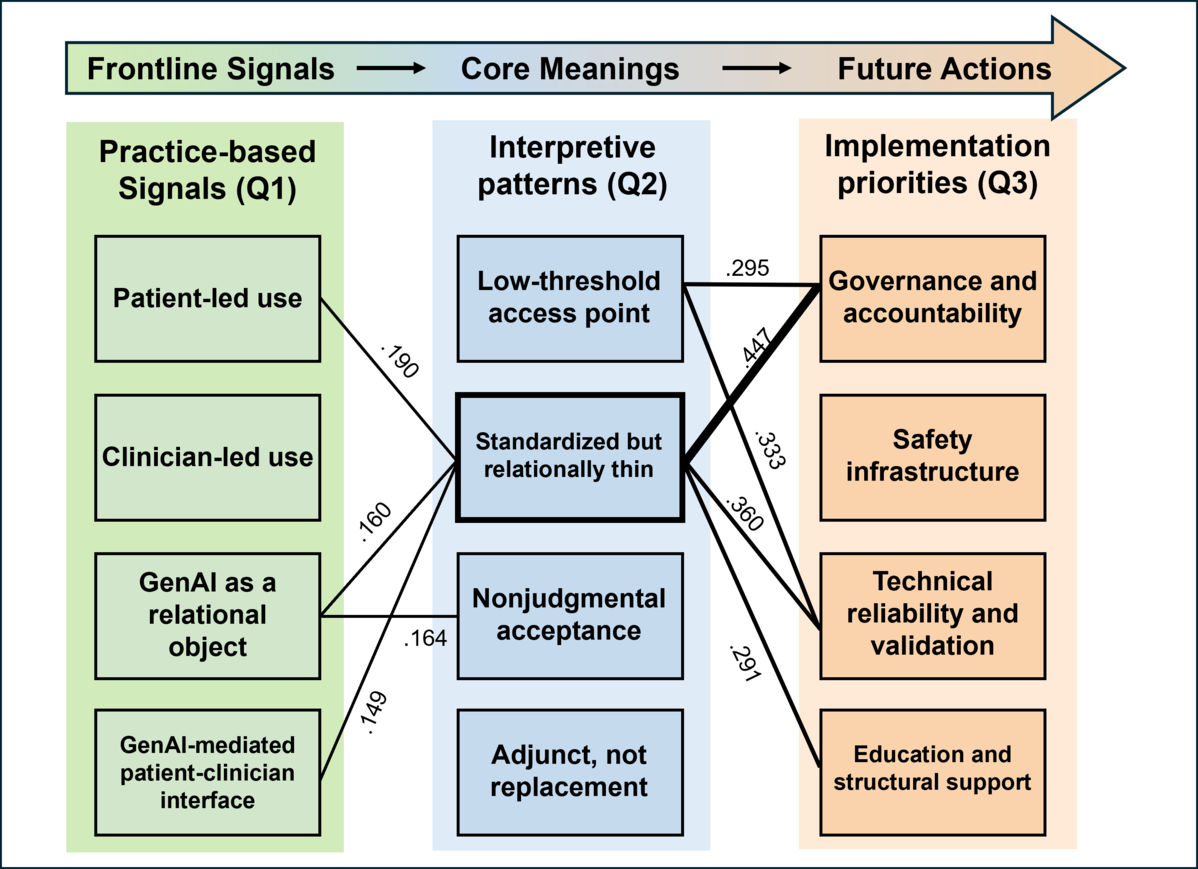
Selected salient alignments across practice-based signals (**Q1**), interpretive patterns (**Q2**), and implementation priorities (**Q3**). Lines indicate theme pairings highlighted from the Results section, based on interpretive salience and relative overlap. Full co-occurrence matrices and Jaccard coefficients are reported in Tables S1 and S2 in Multimedia Appendix 2. GenAI: generative artificial intelligence.

Accessibility as a low-threshold and always-available point of contact formed an important secondary bridge, showing notable overlap with technical reliability and clinical validation before scale-up (Jaccard=0.333) and governance and accountability as prerequisites for adoption (Jaccard=0.295; Table S2 in Multimedia Appendix 2). Taken together, these patterns suggest recurrent reinforcing alignments between frontline signals and perceptions of relational thinness. They also show how interpretive concern bridged to governance- and validation-oriented priorities. To complement the full pairwise matrices in Multimedia Appendix 2, Figure 2 visually synthesizes the most interpretively salient alignments across Q1, Q2, and Q3.

## Discussion

### Principal Findings

This qualitative descriptive study used directed content analysis and codebook-based thematic synthesis, informed by horizon scanning concepts, to examine how psychiatrists in South Korea connected frontline experiences, interpretive framings, and implementation priorities regarding GenAI in psychiatric practice. Rather than documenting attitudes toward GenAI in the abstract, the study examined how psychiatrists linked concrete clinical encounters with broader judgments about the role, limits, and future governance of GenAI in psychiatric care. Across the 3 analytic domains, psychiatrists did not describe GenAI in uniformly positive or negative terms. Instead, they portrayed it as a clinically ambivalent technology: useful as a low-threshold access point and bounded support tool but potentially destabilizing when it entered domains requiring reality testing, crisis containment, or relationship-based therapeutic judgment.

The exploratory cross-question thematic alignment analysis suggested recurrent within-respondent links between practice-based signals, an interpretive view of GenAI as standardized and tireless but relationally thin, and implementation priorities focused on governance and validation. Across domains, the clearest convergence centered less on enthusiasm for innovation than on the conditions required for legitimate introduction into psychiatric care, especially clearer boundaries, stronger governance, and more robust clinical validation for patient-facing use [3,9,11,12,17]. This pattern is consistent with the broader literature, which suggests that the rapid expansion of GenAI in mental health has outpaced the clinical validation, structured oversight, and risk-stratified safeguards needed for psychiatric use [22,37].

### Relational Thinness, Nonjudgmental Acceptance, and Psychiatric Vulnerability

One clinically meaningful pattern concerned how psychiatrists understood GenAI use in the context of patient vulnerability, especially where psychosis, suicidality, or symptom-related overuse were present. In Q1, psychiatrists reported concrete experiences of delusion reinforcement, excessive validation, symptom-linked overuse, and suicide- or self-harm-related concerns. In Q2, these concerns reappeared as broader interpretive worries about nonjudgmental or overly accommodating responses, psychosis-specific vulnerability, and the inability of chatbots to manage crises safely. In Q3, they were reflected in strategic priorities such as psychosis-sensitive safeguards, crisis escalation pathways, misuse prevention, and clearer accountability for harm. The appendix matrices further suggest that this pattern is not reducible to risk alone: relationally salient patient experiences also aligned with the interpretive theme of nonjudgmental acceptance, implying that the same continuously supportive stance that may feel containing in low-risk contexts can become destabilizing under conditions of psychiatric vulnerability. These findings suggest what may be described as a vulnerability amplification pattern, in which features that appear supportive in lower-risk contexts may become destabilizing in the presence of impaired reality testing, compulsive reassurance seeking, severe affective dysregulation, or suicidal intent [38].

This interpretation extends current concerns in the literature in two ways. First, it is consistent with recent commentaries and case-based reports warning that GenAI may reinforce psychotic thinking or delusion-like beliefs in susceptible users [16,17], while also resonating with broader work on emotional dependence and psychosocial deterioration during extended chatbot use [4,38]. Second, it goes beyond prior clinician attitude surveys by grounding these concerns in reported clinical encounters rather than abstract opinion alone [3,11,12]. In this sense, the risks identified here are not best understood as generic AI safety problems arising in psychiatric settings. Rather, they appear to be shaped by psychiatric vulnerability itself. This has important implications for governance: mental health GenAI may require more stratified oversight, diagnosis-sensitive contraindications, and higher thresholds for unsupervised use than many other forms of medical AI [6,22].

### Access-Protection Tension and the Implementation Gap

A second major finding was the tension between perceived accessibility and the absence of a reliable floor of clinical protection. In Q1, psychiatrists described patients using GenAI for self-help, symptom organization, emotional ventilation, and even treatment entry. In Q2, these same functions were reflected in themes of accessibility, immediacy, anonymity, and low-threshold engagement. Yet these benefits did not translate into broad endorsement of patient-facing use. Instead, respondents repeatedly emphasized monitoring difficulty, accountability gaps, and context-sensitive risks that became more salient when GenAI was used beyond limited support functions. Q3 clarified what respondents viewed as missing: practical guidelines, legal and ethical frameworks, expert supervision, privacy protections, and safety systems for high-risk situations. The cross-question alignment matrices sharpened this pattern by highlighting that accessibility aligned more closely with technical validation and governance priorities than with a simple logic of expansion, suggesting that psychiatrists understood access-related benefits as contingent on stronger protective infrastructure rather than as sufficient grounds for wider deployment.

This pattern may be understood as an access-protection tension: current GenAI chatbots and related GenAI tools can lower the threshold of entry into support, but often without a reliable floor of clinical protection. This tension may be particularly relevant in South Korea, where digital uptake is high and psychiatric help-seeking has been shaped by stigma as well as structural concerns related to psychiatric records, employment disadvantage, and other forms of institutional discrimination [20,21,39]. In this context, the appeal of immediate and anonymous AI support is understandable, although these structural factors were not directly elicited in the present survey. However, the present findings suggest that access alone is not an adequate proxy for safe care. The Korean regulatory environment is moving toward stronger oversight of high-impact AI, and recent World Health Organization guidance has likewise emphasized the need for governance, transparency, risk management, and human accountability in the use of large multimodal models for health [22]. Our findings suggest that mental health remains a domain in which this regulatory turn has not yet been translated into sufficiently specific clinical guardrails, particularly for patient-facing systems that are experienced as accessible before they are experienced as clinically protected.

### Conditional Acceptance and Professional Reconfiguration

A third major finding was that psychiatrists’ acceptance of GenAI was conditional rather than oppositional. The results do not support a simple narrative of professional resistance. In Q1, respondents described bounded and pragmatic uses of GenAI in documentation, summarization, medication-related queries, patient communication, and between-visit organization of clinical material. In Q2, these experiences aligned with recognition of AI’s usefulness for accessibility, standardization, and information support. Yet this did not lead to endorsement of GenAI as a substitute therapist. Instead, respondents repeatedly framed GenAI as acceptable mainly when used as an adjunct under bounded indications, and unacceptable when it displaced human therapeutic judgment, contextual formulation, or crisis management. In Q3, this conditional acceptance was mirrored in calls for validation, supervision, quality control, and explicit scope-setting. Beyond the present data, implementation may also require careful communication as well as technical validation, because emerging evidence suggests that disclosure of physician AI use can reduce perceived competence, trustworthiness, and empathy among members of the public [18]. The alignment analysis adds nuance to this conditional acceptance by suggesting that the interpretive theme most consistently related to future priorities was not accessibility alone, but the recognition that GenAI could function in a standardized and reliable manner while remaining relationally thin.

At an interpretive level, this pattern is also consistent with a technology-acceptance perspective, in which perceived usefulness and perceived threat may operate simultaneously rather than sequentially [40,41]. Psychiatrists appeared to recognize usefulness for bounded functions, especially administrative, informational, and preparatory tasks, while withholding support for higher-autonomy, patient-facing substitution. In this sense, the strongest within-respondent pattern was not from usefulness to adoption, but from perceived relational limitation to demands for governance, validation, and clearer boundary-setting. This reading is consistent with earlier studies showing that clinicians tend to be more receptive to GenAI for administrative efficiency, information processing, and standardized support than for empathy-dependent or relationship-centered clinical work [3,11-13].

At the same time, the present results suggest something more than selective task acceptance. They may also indicate an emerging reconfiguration of psychiatric professionalism, in which psychiatrists may increasingly be positioned not only as direct providers of care but also as interpreters, boundary-setters, and safety supervisors of patient-AI interactions. Related qualitative work further suggests that physicians expect GenAI to influence the therapeutic relationship in both supportive and disruptive ways, particularly through altered shared decision-making, transparency expectations, and concerns about the loss of empathic nuance [8]. Because consistent training-level contrasts did not emerge clearly enough in the present qualitative material to support a strong subgroup argument, future work should examine more directly whether these patterns vary by career stage, degree of GenAI exposure, or clinical responsibility. This question remains important given broader evidence from mental health practice showing substantial familiarity gaps, low rates of formal training, and uneven adoption intentions across application types [15].

### Methodological Contribution: Adapting Horizon Scanning Concepts to Practice-Based Signal Mapping

This study also makes a methodological contribution by showing how selected concepts from horizon scanning can be adapted to the qualitative analysis of clinician-reported practice-based signals. Traditional horizon scanning is well suited to identifying external signals from publications, policy developments, patents, and market activity, whereas open-ended clinician reports can capture weak signals emerging within routine care before they are stabilized as formal evidence or policy objects [9,42]. In this study, horizon scanning concepts did not function as a standalone foresight method. Rather, they served as an organizing lens within a qualitative descriptive design for linking frontline clinical signals, interpretive patterns, and implementation priorities. Two elements were adapted in particular: first, the notion of practice-based signals as clinically consequential early indicators emerging within routine care; and second, a 3-layer organizing heuristic linking signal detection, interpretive framing, and priority-setting. The exploratory appendix matrices illustrate one way of operationalizing this linkage without displacing qualitative interpretation: quantified overlap and directional alignment were used not to replace thematic synthesis, but to identify which cross-item pairings warranted closer interpretive attention. In that sense, the contribution of this study lies less in proposing a new futures method than in demonstrating how practice-based clinical experience can serve as a complementary signal source within existing qualitative approaches to future-oriented research in psychiatry.

### Limitations

This study has several limitations. First, the qualitative data came from open-ended survey items rather than interviews or focus groups. Many responses were brief and contextually sparse, which limited interpretive depth. The classification of experiential proximity in Q1 also relied on textual cues, requiring some interpretive judgment when firsthand versus secondhand experience was not explicitly stated.

Second, the cross-question thematic alignment analysis identified recurrent within-respondent pairings across adjacent items. These should be interpreted as descriptive cross-item alignments within a cross-sectional design, not as evidence of temporal sequencing, causal ordering, or inferential association.

Third, the study relied on psychiatrists’ reports of patient-AI encounters and did not independently verify the underlying chatbot interactions, prompts, or platforms involved. The findings, therefore, reflect clinically meaningful professional judgments about AI-related events, rather than model-specific performance. In addition, because the data came exclusively from psychiatrists, the analysis foregrounds clinician-facing concerns and does not directly capture patient perspectives on the accessibility, acceptability, or harms of AI-based support.

Fourth, the thematic alignment matrices used item-level theme frequencies and pairwise cross-item co-occurrence counts to characterize how respondents endorsing a given theme were distributed across themes in adjacent items. These indicators were useful for exploratory pattern detection, although they remain sensitive to differences in base theme frequency and should not be interpreted as exact estimates conditional on response to both items. Because the main text emphasized Jaccard coefficients as prevalence-adjusted summaries, some directionally informative detail was shifted to the appendix matrices, which report full co-occurrence counts and conditional proportions.

### Conclusions

GenAI appears to be entering psychiatric practice as an access tool, a workflow aid, and, at times, a competing interpretive influence within clinical encounters. The central challenge is not whether psychiatry will encounter GenAI, but how its use should be bounded, supervised, and governed, considering the vulnerabilities, relational demands, and safety risks specific to psychiatric care. These findings suggest that implementation should prioritize bounded adjunctive use, stronger governance and validation, and mental health-specific oversight rather than rapid substitution of human care.

## Supplementary material

10.2196/96556 Multimedia Appendix 1 Qualitative codebook, code definitions, respondent-level frequencies, and illustrative quotations.

10.2196/96556 Multimedia Appendix 2 Within-respondent thematic alignments across experience, interpretation, and priority.

## Abbreviations

AI: artificial intelligence
GenAI: generative artificial intelligence
