# Supplementary material for: Mapping Practice-Based Signals of Generative AI in Psychiatric Care: Qualitative Study of Korean Psychiatrists’ Experiences, Interpretations, and Implementation Priorities
Source: J Med Internet Res. 2026 Jun 2;28:e96556. doi: 10.2196/96556 (PMC13229467; doi:10.2196/96556)
Supplement: Multimedia Appendix 1 [file jmir-v28-e96556-s001.docx]

**Multimedia Appendix 1. Qualitative codebook, code definitions, respondent-level frequencies, and illustrative quotations.**

This appendix presents the full codebook used in the directed content analysis of responses to the three open-ended survey questions. Codes are organized by question and listed in descending order of frequency. For Q2, codes are grouped by category (advantages and limitations of AI chatbots relative to human therapists). Each entry includes the code label, operational definition, frequency as a proportion of question-level respondents (Q1: n = 218; Q2: n = 232; Q3: n = 220), and a representative quote drawn directly from survey responses. A single response could receive more than one code when distinct content was present. Quotes were translated from Korean into English and lightly edited for readability while preserving the original meaning. Respondent identifiers are indicated in parentheses. This appendix also provides a code-to-theme mapping table for each question, indicating which codes were grouped under each theme during thematic synthesis. Theme labels follow the manuscript narrative exactly.

**Inter-rater Reliability**

Q1: In the initial independent coding, the mean Cohen's κ was 0.643. While some codes demonstrated high agreement from the outset (κ ≥ 0.80), lower agreement was observed for others. Through iterative discussion and refinement of code definitions, the Clinician_learning_teaching code was removed, and two new codes, Material_development_aid and Ai_information_seeking, were added in its place. Following recoding, most codes improved to κ ≥ 0.61, and final coding was confirmed through consensus.

Q2: Initial coding revealed substantial variability in Cohen's κ across codes, with some codes showing high agreement and others demonstrating low agreement (e.g., Psychosis_delusion_risk, κ = 0.3803). Iterative refinement was conducted, including clarification of code definitions and structural reorganization, followed by two rounds of recoding. As a result, most codes reached Cohen's κ ≥ 0.61 in the final coding, and codes that had initially shown low agreement were substantially improved (e.g., Psychosis_delusion_risk, κ = 0.7690; Crisis_response_failure, κ = 0.9452). Final coding was confirmed through consensus.

Q3: Inter-rater agreement for Q3 was generally strong from the initial independent coding. Six of the 16 codes (Privacy_data_protection κ = 0.9684, Ethics_framework κ = 0.9312, Expert_supervision κ = 0.9290, Accountability_liability κ = 0.8962, Reimbursement_incentive κ = 0.8704, Education_awareness κ = 0.8616) achieved κ ≥ 0.80 from the outset, and the remaining nine codes also met the threshold of κ ≥ 0.61, indicating substantial agreement. Only Scope_indication (κ = 0.5650) fell below this threshold at the moderate level. A focused discussion was conducted to clarify its definition and reduce interpretive ambiguity, after which final coding was confirmed through consensus.

**Table S1. Practice-Based Signals from Clinical Encounters with Generative AI (Q1)**

| **Code** | **Definition** | **n (%)** | **Representative Quote** |
| --- | --- | --- | --- |
| PATIENT_ SELFHELP | Patient voluntarily uses AI as a self-help tool for psychological difficulties or emotion regulation, reporting positive effects. | 43 (19.72%) | “A patient with a strong desire for therapeutic treatment chatted with AI every day, building their own psychodynamic formulation and bringing it to sessions. It was impressive that they were also seeing a private counselor, so the review process between clinicians likely contributed to the outcome.” (P195) |
| MEDICATION_ INFO_USE | Patient or clinician consults AI about medication information, side effects, or drug interactions. Includes both helpful and harmful outcomes. | 24 (11.01%) | “I have experience using ChatGPT to look up drug interactions and fill gaps in my medical knowledge. When prompts are written well, AI hallucinations decrease, and double-checking the facts significantly reduces the time spent searching for information.” (P270) |
| CLINICIAN_ CLINICAL_DECISION_AID | Clinician uses AI to support clinical reasoning: differential diagnosis, medication selection, test interpretation, or case summarization. | 23 (10.55%) | “Looking at an AI psychiatric diagnostic support tool, I felt frustrated; what productivity benefit is there in having AI diagnose from records already written by the psychiatrist? If the tool could observe the session via camera and detect changes in the patient’s expressions and speech to support diagnosis, that would have been truly groundbreaking.” (P112) |
| PRE_ CONSULTATION_AID | Patient uses AI to recognize symptoms or is advised by AI to seek psychiatric care; AI functions as a treatment 'gateway'. | 21 (9.63%) | “A patient initially seen for depression chatted with a chatbot about symptom changes during antidepressant treatment. The chatbot suggested a possible bipolar disorder and recommended consulting a physician. The patient came in earlier than scheduled, bringing a chatbot-generated summary; it was clinically valid, and the patient stabilized after starting bipolar treatment.” (P361) |
| PATIENT_ AI_SHARING | Patient brings AI-generated conversation logs or symptom summaries to a clinician; AI functions as an information intermediary. | 18 (8.26%) | “A patient with a strong desire for therapeutic treatment chatted with AI every day, building their own psychodynamic formulation and bringing it to sessions. I found this impressive as a way to make the most of limited clinic time.” (P195) |
| DELUSION_ REINFORCEMENT | AI interaction consolidates existing delusions, induces new delusions, or otherwise worsens psychotic symptoms. | 16 (7.34%) | “I have experienced cases in clinical practice where psychotic patients’ delusions deepened through chatbot conversations. Pre-existing delusions became consolidated, and in many cases new delusional content and thinking were added as a result of AI interactions.” (P44) |
| TREATMENT_ NONCOMPLIANCE | Patient, based on AI recommendations, refuses clinician instructions, arbitrarily changes medications, or conceals symptoms, undermining therapeutic cooperation. | 12 (5.5%) | “There were cases where patients searched for information through AI and developed a negative perception of the treatment process or learned ways to mask their symptoms from clinicians. For example, a patient looked up side effects of their prescribed medication, then reported those side effects and refused to continue taking the drug.” (P182) |
| AI_INFORMATION_ SEEKING | Patient or clinician uses AI specifically to look up medical or health-related information. | 12 (5.5%) | “I have experience using ChatGPT to look up drug interactions and fill gaps in my medical knowledge. When prompts are written well, AI hallucinations decrease, and double-checking the facts significantly reduces the time spent searching for information.” (P270) |
| HALLUCINATION_ MISINFORMATION | Clinician experiences AI providing factually incorrect, contextually inappropriate, or expectation-distorted information. | 11 (5.05%) | “There were cases where patients asked a generative AI about their condition or the side effects of their medications and came to believe incorrect information, which was a concerning pattern.” (P137) |
| SOCIAL_ISOLATION_ FUNCTIONING | AI use leads to changes in socially isolated patients. Includes both positive changes (improved communication) and negative changes (deepened isolation). | 11 (5.05%) | “A patient with Asperger’s who talked to themselves a lot found that chatting with a chatbot helped pass the time and reduced disturbance to others, satisfying both the patient and those around them.” (P261) |
| PATIENT_CLINICIAN_COMPARISON | Patient explicitly compares clinician's advice against AI responses, or uses AI output as a benchmark to evaluate the clinician. | 11 (5.05%) | “In everyday conversations with the general public about medical topics, there were cases where people trusted AI information more than my own words as a physician. This was more pronounced among those with lower levels of education.” (P25) |
| ADMIN_TASK_AID | Clinician uses AI for administrative tasks requiring no clinical judgment: documentation, translation, summarization, or patient instruction generation. | 11 (5.05%) | “At a multidisciplinary consultation meeting, I used an AI-based tool to quickly generate a clinical summary related to the patient’s psychiatric condition. AI summarized key information from medical records, making the consultation process more systematic and efficient.” (P388) |
| AI_ OVERDEPENDENCE | Patient or layperson uses AI excessively in time or frequency, or cannot function daily without it. | 10 (4.59%) | “A Bipolar I patient continued to use the shared computer on the ward to access GPT. When we tried to limit usage time, the patient acted out. The patient believed GPT could help them become a billionaire through cryptocurrency.” (P13) |
| SUICIDE_SELFHARM | AI interaction is directly or indirectly associated with suicide or self-harm—including provision of method information, suicide attempt linkage, or crisis intervention. | 10 (4.59%) | “There were cases where patients obtained information about suicide methods through chatbots. Given the difficulty of detecting the user’s intent, a control program to address this is needed.” (P213) |
| SPECIFIC_AI_TOOL_ EXPERIENCE | Clinician shares personal experience or opinion about a specific AI tool (e.g., Open Evidence). | 10 (4.59%) | “Looking at an AI psychiatric diagnostic support tool, I felt frustrated about whether it actually improves productivity when AI diagnoses from records already written by the psychiatrist. A camera-based tool that could detect changes in the patient’s expressions and speech during the session would have been truly groundbreaking.” (P112) |
| EXCESSIVE_ VALIDATION | AI excessively agrees with a patient or provides unconditional reinforcement, exhibiting a clinically inappropriate supportive response. | 10 (4.59%) | “Some patients reported that AI advised them to visit a psychiatric hospital, while others said AI just unconditionally supported and reassured them that everything was fine. I believe there is a need to raise public awareness about avoiding uncritical use of AI.” (P12) |
| PATIENT_ AI_RELIANCE | Patient perceives AI as a substitute for human relationships or as a primary emotional support, with emphasis on closeness or feeling understood. | 9 (4.13%) | “A first-visit patient said that chatting with AI for several hours every night over the past three years had been the most helpful experience, more than any clinic or counselor they had tried. They came to hear additional perspectives.” (P65) |
| AI_MISUSE_ DECEPTION | Patient uses AI to intentionally distort or conceal symptom information, or an AI-based self-diagnosis is incorrect and harms the treatment process. | 7 (3.21%) | “There were cases where patients used AI to learn how to mask their symptoms from clinicians. For example, a patient looked up side effects of their prescribed medication, then reported those side effects and refused to take the drug.” (P182) |
| SYMPTOM_ TRIGGERED_AI_USE | Patient's AI use increases as mental health worsens; symptom exacerbation and excessive AI use are mutually reinforcing. | 6 (2.75%) | “In most cases involving patients with obsessive-compulsive disorder, symptoms deteriorate sharply after using AI chatbots. I now routinely recommend discontinuing AI chatbot use as part of treatment.” (P241) |
| MENTAL_HEALTH_ DETERIORATION | Patient's non-psychotic mental health (anxiety, obsessive thoughts, mood, dysfunctional thinking) deteriorates after AI use. | 6 (2.75%) | “In most cases involving patients with obsessive-compulsive disorder, symptoms deteriorate sharply after using AI chatbots. I now routinely recommend discontinuing AI chatbot use as part of treatment.” (P241) |
| CLINICIAN_AI_ APPROPRIATENESS_ CHECK | Patient asks a clinician to confirm whether a specific AI use is clinically appropriate. | 6 (2.75%) | “A patient submitted a summary of their chatbot conversations at the outpatient clinic. They also asked whether it would be possible to prescribe the medication that ChatGPT had recommended for their symptoms.” (P211) |
| MATERIAL_ DEVELOPMENT_AID | AI is used to create educational materials, patient handouts, manuals, or similar content. | 6 (2.75%) | “One memorable experience was using a chatbot to create explanatory handouts for patients about their test results. In mental health, results are often presented in technical terms that patients find difficult to understand. I used the chatbot to summarize MMPI, BDI, and STAI results in everyday language.” (P388) |
| AI_AS_CONSULT | Clinician seeks clinical consultation from AI instead of making a formal referral to a specialist. | 5 (2.29%) | “I personally use AI when I am curious about conditions outside my specialty. Once I took time off work and brought my child to the hospital based on AI saying it was an emergency; it turned out to be nothing serious. It seems AI has been trained to flag situations as urgent.” (P13) |
| PATIENT_ BEST_KNOWN_AI | Patient or clinician perceives AI as the entity that best understands their condition, or patient reports a substantially large therapeutic effect through AI. | 5 (2.29%) | “A first-visit patient said that chatting with AI for several hours every night over the past three years had been the most helpful experience, as though AI understood them better than any therapist they had seen.” (P65) |
| MISDIAGNOSIS_ TRUST_DECLINE | AI makes a clinically inappropriate diagnosis or recommendation, causing real decline in AI trust and directly affecting the patient-clinician relationship or treatment. | 4 (1.83%) | “A patient came to me angry after telling ChatGPT their symptoms and receiving an answer about the diagnosis, saying that their attending psychiatrist had given a different diagnosis and that they could no longer trust the attending physician. In the end, the attending psychiatrist’s diagnosis was correct, and the problem had arisen because the patient mentioned only biased symptoms, that is, only those they themselves acknowledged.” (P53) |
| CLINICIAN_PATIENT_AI_DISCUSSION | AI-related topics arise naturally during a clinical consultation; AI appears as a topic within the clinical context. | 4 (1.83%) | “In the mental health clinic, I consulted a conversational AI about the patient’s medical issues and discussed it with the patient. Patient satisfaction was high.” (P66) |
| CLINICIAN_ PERSONAL_USE | Clinician uses AI to manage their own occupational stress, personal concerns, or psychological burnout. | 2 (0.92%) | “After an argument with my wife, on my way to work I vented to a chatbot using voice input. The AI’s response felt so human-like that I experienced a genuine sense of emotional release.” (P376) |
| INAPPROPRIATE_AI_ SERVICE | Patient is exposed to clinically unverified or inappropriate AI-related services outside mental health settings. | 1 (0.46%) | “In clinical practice I frequently observe AI services used outside mental health contexts affecting mental health. For example, adolescents are being indiscriminately exposed to sexually distorted characters in AI fan fiction writing platforms. This is less a problem of the technology itself than of the service platform policy.” (P151) |
| FAKE_AD_ FACTCHECK | Patient uses AI as a fact-checking tool to avoid being misled by exaggerated medical advertisements. | 1 (0.46%) | “An increasing number of patients are being misled by fake AI doctor advertisements and exaggerated medical claims on YouTube. I now advise them not to trust such content and instead to get information from ChatGPT.” (P190) |

**Table S2. Interpretive Themes Regarding AI Chatbots in Mental Health Care: Advantages and Limitations (Q2)**

| **Code** | **Definition** | **n (%)** | **Representative Quote** |
| --- | --- | --- | --- |
| **Advantages** | | | |
| ACCESSIBILITY | Available anytime, anywhere without geographic or temporal barriers (including 24/7 availability). | 85 (36.64%) | “AI chatbots have the advantage of immediacy and accessibility. Therefore, they should be understood not as therapeutic replacements, but as entry points for those who cannot access treatment and as tools that support human care.” (P33) |
| INFORMATION_ PROVISION | Provides rich, rapid information based on vast medical knowledge and big data, including up-to-date drug interactions. | 44 (18.97%) | “Immediacy, rapid presentation of diverse alternatives, and fast analysis are advantages. However, it is not easy for patients to trust AI chatbots enough to reveal themselves truthfully, as they would with a reliable human therapist.” (P230) |
| NO_ COUNTERTRANSFERENCE | Delivers consistent service without countertransference, burnout, or emotional variability. | 33 (14.22%) | “Reliability and tirelessness, always being responsive, are the biggest advantages. However, these same qualities can become disadvantages: patients may not develop the ability to cope on their own, and they may become accustomed to abusive language that would not be tolerated in real human relationships.” (P22) |
| CONSISTENCY_ STANDARDIZATION | Provides consistent, standardized responses regardless of therapist competence variability; supports uniform evidence-based protocols. | 30 (12.93%) | “A major advantage is that AI chatbots can provide counseling with a guaranteed level of quality, unlike human therapists whose quality varies considerably, and the negative effects of transference and countertransference can also be eliminated.” (P134) |
| OBJECTIVITY_ NEUTRALITY | Responds objectively and neutrally, free from human therapists' subjective biases. | 14 (6.03%) | “Quickly acquiring large amounts of information and maintaining neutrality are advantages; the absence of emotion is useful here. However, being non-human means genuine empathy and understanding of patients will be lacking.” (P408) |
| ANONYMITY_ REDUCED_STIGMA | Guarantees anonymity and reduces stigma, enabling disclosure of sensitive content. | 13 (5.6%) | “An advantage is that it can reduce the psychological burden for patients who find it difficult to express their emotions to others. A limitation is that integrated adjustment of feedback is difficult; it may be hard to intervene when some feedback is distorted.” (P216) |
| COST_EFFICIENCY | Available at low or no cost compared to human therapists, reducing economic burden. | 13 (5.6%) | “Advantages include reduced transference/countertransference and benefits in terms of time and cost. Limitations include constraints arising from the perception that it is not a human relationship, and limitations in grasping social context.” (P89) |
| ADJUNCTIVE_ UTILITY | Useful as a supplement to human therapists rather than as an independent therapeutic agent. | 12 (5.17%) | “In conclusion, AI chatbots are most effective when used as a complement to, rather than a replacement for, human therapists.” (P388) |
| IMMEDIACY | Responds instantly to user requests; capable of immediate intervention in crisis moments. | 11 (4.74%) | “Chatbots have an advantage in that patients can engage 24/7 instantly at any time. However, I believe humans crave interaction with other people, so AI alone cannot meet all patients’ needs. Chatbots should be used for patients to describe and reflect on their thoughts, with therapists reviewing and supervising the content.” (P111) |
| TREATMENT_ ENTRY_POINT | Lowers barriers to mental health treatment; supports mild cases and facilitates treatment initiation. | 6 (2.59%) | “AI is expected to rapidly improve the efficiency of drug prescribing, drug interactions, and cognitive therapy. However, the final decision and responsibility, and sharing emotions, will remain the province of the human physician.” (P224) |
| UNCONDITIONAL_ ACCEPTANCE | Offers unconditional, continuous acceptance and support; provides psychological safety without judgment. | 6 (2.59%) | “Patients who feel depressed or isolated late at night and chat with GPT to feel stable; I have encountered countless such cases. AI can serve well as a stabilizing agent. However, because it tends to give only positive answers, patients sometimes need to specify ‘respond coldly’ or ‘accurately’ in their prompts.” (P195) |
| **Limitations** | | | |
| CONTEXT_ INDIVIDUALIZATION_LIMIT | Limited ability to understand individual patient characteristics, medical history, cultural context, and provide truly individualized responses. | 39 (16.81%) | “Being able to transcend spatial and temporal constraints is an advantage. However, I am cautious about whether AI can respond appropriately to the individual characteristics of each patient or to the current pathological state and severity, especially in psychiatric emergencies.” (P53) |
| HALLUCINATION_ INACCURACY | Exhibits hallucination phenomena and general inaccuracy, providing unreliable or false information. | 30 (12.93%) | “Although the hallucination problem has improved, the ability to distinguish psychiatric emergencies remains poor, and providing a false sense of security to patients in urgent situations is a major concern.” (P69) |
| LACK_OF_EMPATHY | Lacks the emotional empathy, human connection, and warmth intrinsic to human therapeutic relationships. | 27 (11.64%) | “I personally believe that humans crave interaction with other people, and AI alone cannot meet all patients’ needs. Chatbots are best used for patients to freely describe and reflect on their thoughts, with the therapist then reviewing the content.” (P111) |
| NONVERBAL_ COMMUNICATION_ LIMIT | Unable to capture or interpret nonverbal cues such as facial expressions, gestures, eye contact, or vocal tone. | 25 (10.78%) | “Because evaluation is focused on the text the person expresses, it may be difficult to read between the lines, catching nuance, dishonest expressions, or exaggerated emotions. General counseling for the average person is possible, but in severely ill psychotic states this could cause serious problems.” (P178) |
| THERAPEUTIC_ RELATIONSHIP_ IRREPLACEABLE | Cannot replicate therapeutic change through the human therapeutic relationship, transference analysis, or intersubjectivity. | 22 (9.48%) | “A limitation is that patients are unlikely to trust AI chatbots enough to truly reveal themselves as they would with a reliable human therapist. For psychoanalytic treatment, transference analysis, and achieving genuine inner change through intersubjectivity, chatbots have clear limitations.” (P230) |
| ALIGNMENT_ SYCOPHANCY | Provides only answers users want to hear; lacks critical feedback or therapeutic challenge. | 20 (8.62%) | “While a human therapist approaches content the patient does not want to discuss, AI still tends to be passive and operates mainly to accommodate the patient’s needs. Attention to this kind of bias is needed, especially in cases involving patients with delusional disorder where chatbot use may reinforce symptoms.” (P67) |
| LEGAL_ETHICAL_ ACCOUNTABILITY | Legal and ethical responsibility is unclear or absent; raises personal data protection concerns. | 12 (5.17%) | “Ultimately, the biggest issue will be who bears responsibility. This is similar to the situation with Tesla’s autonomous driving, which drives better than human drivers in most cases, has not been easily adopted. The same fundamental accountability problem applies to AI in clinical care.” (P112) |
| DEPENDENCY_ SOCIAL_ WITHDRAWAL | Excessive AI use reinforces avoidance of real human relationships, leading to regression in social functioning. | 11 (4.74%) | “Reliability and tirelessness are the biggest advantages. But these same qualities can also become disadvantages; patients may not develop the ability to cope on their own. Another concern is that patients may become accustomed to abusive language that would not be tolerated in real human relationships.” (P22) |
| PSYCHOSIS_ DELUSION_RISK | Accommodating responses may reinforce delusions in patients with psychosis or impaired reality testing. | 10 (4.31%) | “AI still tends to be passive and operates mainly to accommodate the patient’s needs rather than challenging them. This bias is particularly concerning in recent cases of delusional disorder patients using chatbots, where symptoms such as delusions appear to have been reinforced.” (P67) |
| CRISIS_RESPONSE_ FAILURE | Inadequate in responding to emergencies such as suicide or self-harm; risk of false sense of security. | 10 (4.31%) | “I am cautious about whether AI can respond appropriately to the current pathological state and severity of each patient. Particularly in psychiatric emergencies, there is no clear solution for how AI should respond and manage the situation.” (P53) |
| SUPERVISION_ MONITORING_ DIFFICULTY | Difficult for professionals to supervise or monitor AI conversation content and clinical judgments. | 6 (2.59%) | “Chatbots should be used for patients to freely describe and reflect on their thoughts, with therapists reviewing and supervising the content to prevent impairment of reality testing. However, it is difficult for clinicians to monitor what patients discuss with AI and how they use that content.” (P111) |

**Table S3. Implementation Priorities for the Safe Introduction of Generative AI into Mental Health Care (Q3)**

| **Code** | **Definition** | **n (%)** | **Representative Quote** |
| --- | --- | --- | --- |
| ETHICS_ FRAMEWORK | Establishing AI ethical standards: ethics codes, application of medical ethics principles, harm prevention, and ethics-based usage standards. | 49 (22.27%) | “There should be a basic code of ethics for AI technology, along with practical guidelines and a supervisory body to ensure that it is used appropriately.” (P32) |
| GUIDELINE_LEGAL_ FRAMEWORK | Preparing usage guidelines and legal/institutional frameworks: academic society/government guidelines, legal regulations, and clinical manuals. | 45 (20.45%) | “When enquiries come in from the clinic, it would be helpful to have guidelines at the society level that explain the use of AI to patients, covering points such as when its use is beneficial and when it should not be used.” (P49) |
| INFORMATION_  QUALITY | Ensuring information accuracy and reliability: evidence-based training data, source citation, and inaccurate information regulation. | 40 (18.18%) | “There needs to be a way to address hallucinations so that the system responds only on the basis of evidence-based materials.” (P82) |
| PRIVACY_DATA_ PROTECTION | Protecting personal and clinical data: security of sensitive information, consent for training data, and prevention of information leakage. | 39 (17.73%) | “It is necessary to define the anticipated limitations and problems of AI in clinical care and share awareness of these to prevent misuse. When users’ sensitive information is entered during AI use, safeguards must be in place to prevent personal data leakage and related issues.” (P144) |
| EDUCATION_ AWARENESS | Improving education and awareness: AI literacy for clinicians, guidance on limitations for patients, and public outreach. | 36 (16.36%) | “Both patients and physicians need to use AI with an awareness of its limitations, so related education and oversight at the level of academic societies or government are necessary.” (P177) |
| ACCOUNTABILITY_ LIABILITY | Clarifying legal responsibility: identifying responsible parties for adverse events and defining scope of developer/clinician/platform liability. | 35 (15.91%) | “For therapeutic AI, stricter standards are required, including clear identification of who is accountable and what level of supervision is mandated when harm occurs.” (P361) |
| EXPERT_ SUPERVISION | Building professional monitoring systems: mandatory physician supervision, regular AI response audits, and expert advisory bodies. | 32 (14.55%) | “A body of experts capable of overseeing the use of AI should be established at the academic and national levels.” (P223) |
| HALLUCINATION_ RESOLUTION | Technical improvements to resolve hallucination: detection/correction mechanisms and ensuring fact-based responses. | 30 (13.64%) | “It would be problematic to recommend incorrect treatment methods, such as those involving hallucination.” (P278) |
| CLINICAL_ VALIDATION | Clinical validation of efficacy and safety: clinical trials, comparative studies, and evidence of safety. | 25 (11.36%) | “Verification that AI has been trained on validated data; verification of whether regular audits of AI function and responses are possible; and research on the safety of AI use, including whether it is safe under expert supervision, under some degree of supervision, or without supervision.” (P369) |
| SAFETY_CRISIS_ RESPONSE | Building safety and crisis response infrastructure: suicide/self-harm risk detection, emergency linkage protocols, and high-risk group identification. | 25 (11.36%) | “Institutional safeguards are needed for patient safety, such as automatic reporting to relevant agencies or professionals when warning signs related to self-harm or harm to others are identified.” (P135) |
| MISUSE_ PREVENTION | Preventing misuse and over-trust: restricting indiscriminate use, regulating commercial misuse, and curbing excessive AI over-reliance. | 23 (10.45%) | “Expansion of physician education systems for AI-based medical information updates. Physicians must become mediators who help patients interpret the noise in medical information, and it is also necessary to inform patients of the limitations of that information.” (P254) |
| AI_TECH_ ADVANCEMENT | AI technological development and mental health-specific systems: mental health algorithms, cultural/language adaptation, and personalized responses. | 22 (10.0%) | “Settings involving overfitting to patients or excessive flattery need correction. Since AI is designed to increase usage time, therapeutic AI needs settings that differ substantially from those of general LLMs.” (P111) |
| SCOPE_INDICATION | Defining scope and indications: applicable conditions, target populations, and distinction between supportive vs. therapeutic roles. | 20 (9.09%) | “In the case of AI technology based on language, I believe it would be difficult to accurately interpret the speech of infants and children whose minds are still developing, or that of elderly patients with neurocognitive disorders. Indeed, I believe it could distort their speech, potentially harming the development of children and adolescents, or, in the case of patients with neurocognitive disorders, instilling delusions. Therefore, I believe it would be safer to restrict the age range for use to young adults or middle-aged and older adults. I believe a discussion regarding the appropriate age range is essential..” (P44) |
| REIMBURSEMENT_ INCENTIVE | Fee schedules, insurance, and economic support: fee recognition, insurance coverage, and EMR integration support. | 9 (4.09%) | “(1) Investment to strengthen AI capabilities specific to mental health. (2) Creating a professional oversight body. (3) Building consensus on use and regulation, including reimbursement mechanisms and sustainable integration into existing healthcare systems.” (P223) |
| PSYCHOSIS_ SPECIFIC_RISK | Managing risks specific to psychosis and vulnerable populations: delusion reinforcement and AI contraindications for psychotic patients. | 7 (3.18%) | “Setting accountability boundaries between users and developers; overcoming the uncertainty of technical implementation; and an institutional approach to addressing cases where AI clearly causes harm to patients with psychosis or OCD.” (P241) |
| STANDARDIZATION | Standardizing treatment processes: psychotherapy content standardization, AI-assisted treatment protocols, and academic society-led roadmaps. | 6 (2.73%) | “(1) Creating standardized, high-quality treatment materials. (2) Forming a special committee at the academic society level to address AI introduction in mental health and clinical education. (3) Developing a roadmap for mental health-related algorithm development through academic society leadership.” (P27) |

**Table S4. Code-to-Theme Mapping for Q1(Practice-Based Signals from Clinical Encounters with Generative AI)**

| **Theme** | **Codes** |
| --- | --- |
| 1-1. Patient-led signals: self-help, triage, and early gateway use | PATIENT_SELFHELP, PRE_CONSULTATION_AID, FAKE_AD_FACTCHECK, CLINICIAN_AI_APPROPRIATENESS_CHECK |
| 1-2. Clinician-led signals: workflow support, clinical reasoning, and bounded experimentation | CLINICIAN_CLINICAL_DECISION_AID, ADMIN_TASK_AID, MATERIAL_DEVELOPMENT_AID, AI_AS_CONSULT, SPECIFIC_AI_TOOL_EXPERIENCE, CLINICIAN_PERSONAL_USE, HALLUCINATION_MISINFORMATION, AI_INFORMATION_SEEKING, MEDICATION_INFO_USE |
| 1-3. AI as a relational object: attachment, substitution, and social functioning | SOCIAL_ISOLATION_FUNCTIONING, PATIENT_AI_RELIANCE, AI_OVERDEPENDENCE, SYMPTOM_TRIGGERED_AI_USE, EXCESSIVE_VALIDATION |
| 1-4. AI-mediated changes in the patient-clinician interface | PATIENT_AI_SHARING, PATIENT_CLINICIAN_COMPARISON, MISDIAGNOSIS_TRUST_DECLINE, CLINICIAN_PATIENT_AI_DISCUSSION, PATIENT_BEST_KNOWN_AI, TREATMENT_NONCOMPLIANCE, AI_MISUSE_DECEPTION |
| High-risk destabilizing scenarios (cutting across themes) | DELUSION_REINFORCEMENT, SUICIDE_SELFHARM, MENTAL_HEALTH_DETERIORATION, INAPPROPRIATE_AI_SERVICE |

*Note*. High-risk destabilizing scenarios cut across all four themes rather than belonging exclusively to any one theme.

**Table S5. Code-to-Theme Mapping for Q2(Interpretive Themes Regarding AI Chatbots in Mental Health Care)**

| **Theme** | **Codes** |
| --- | --- |
| 2-1. AI as a low-threshold and always-available point of contact | ACCESSIBILITY, IMMEDIACY, COST_EFFICIENCY, TREATMENT_ENTRY_POINT |
| 2-2. Standardized and tireless, but relationally thin | CONSISTENCY_STANDARDIZATION, NO_COUNTERTRANSFERENCE, INFORMATION_PROVISION, LACK_OF_EMPATHY, NONVERBAL_COMMUNICATION_LIMIT, CONTEXT_INDIVIDUALIZATION_LIMIT, ADJUNCTIVE_UTILITY, HALLUCINATION_INACCURACY |
| 2-3. Nonjudgmental acceptance as both comfort and clinical hazard | ANONYMITY_REDUCED_STIGMA, OBJECTIVITY_NEUTRALITY, UNCONDITIONAL_ACCEPTANCE, PSYCHOSIS_DELUSION_RISK, DEPENDENCY_SOCIAL_WITHDRAWAL, CRISIS_RESPONSE_FAILURE, ALIGNMENT_SYCOPHANCY |
| 2-4. Useful as an adjunct, not acceptable as a replacement | THERAPEUTIC_RELATIONSHIP_IRREPLACEABLE, SUPERVISION_MONITORING_DIFFICULTY, LEGAL_ETHICAL_ACCOUNTABILITY |

*Note*. Codes within each theme may include both advantage-oriented and limitation-oriented codes, as Q2 responses were analyzed thematically without separating advantages from limitations.

**Table S6. Code-to-Theme Mapping for Q3(Implementation Priorities for the Safe Introduction of Generative AI into Mental Health Care)**

| **Theme** | **Codes** |
| --- | --- |
| 3-1. Governance and accountability as prerequisites for adoption | ACCOUNTABILITY_LIABILITY, ETHICS_FRAMEWORK, PRIVACY_DATA_PROTECTION, GUIDELINE_LEGAL_FRAMEWORK, REIMBURSEMENT_INCENTIVE |
| 3-2. Safety infrastructures for crisis situations and vulnerable populations | SAFETY_CRISIS_RESPONSE, PSYCHOSIS_SPECIFIC_RISK |
| 3-3. Technical reliability and clinical validation before scale-up | INFORMATION_QUALITY, CLINICAL_VALIDATION, EXPERT_SUPERVISION, HALLUCINATION_RESOLUTION, AI_TECH_ADVANCEMENT, STANDARDIZATION |
| 3-4. Education, supervision, and structural support for responsible use | MISUSE_PREVENTION, EDUCATION_AWARENESS, SCOPE_INDICATION |
