# Supplementary material for: Mapping Practice-Based Signals of Generative AI in Psychiatric Care: Qualitative Study of Korean Psychiatrists’ Experiences, Interpretations, and Implementation Priorities
Source: J Med Internet Res. 2026 Jun 2;28:e96556. doi: 10.2196/96556 (PMC13229467; doi:10.2196/96556)
Supplement: Multimedia Appendix 2 [file jmir-v28-e96556-s002.docx]

**Multimedia Appendix 2.
Within-respondent thematic alignments across experience, interpretation, and priority**

This appendix presents the full cross-question thematic alignment matrices used in the exploratory participant-level analysis. Theme labels are kept consistent with the manuscript narrative. Raw co-occurrence counts are reported alongside a prevalence-adjusted overlap index (Jaccard) and directional conditional proportions. These indicators were used descriptively to support interpretation of cross-item thematic alignment rather than as inferential association measures. Of the 218 Q1 respondents and 232 Q2 respondents, 144 provided interpretable responses to both items and were therefore included in the Q1-Q2 alignment analysis. Of the 232 Q2 respondents and 220 Q3 respondents, 210 provided interpretable responses to both items and were included in the Q2-Q3 alignment analysis. Theme-level respondent counts are not mutually exclusive; a single respondent may be represented in more than one theme within the same question, and theme-level totals therefore exceed the item-level respondent counts.

**Theme labels used in the appendix.** Q1: Patient-led signals (self-help, triage, and early gateway use); Clinician-led signals (workflow support, clinical reasoning, and bounded experimentation); AI as a relational object(Attachment/substitution signals); GenAI-mediated changes in the patient-clinician interface. Q2: GenAI as a low-threshold and always-available point of contact; Standardized and tireless, but relationally thin; Nonjudgmental acceptance as both comfort and clinical hazard; Useful as an adjunct, not acceptable as a replacement. Q3: Governance and accountability as prerequisites for adoption; Safety infrastructures for crisis situations and vulnerable populations; Technical reliability and clinical validation before scale-up; Education, supervision, and structural support for responsible use.

**Table S1. Cross-question thematic alignments between Q1 and Q2**

| **Q1 (nQ1=218)** | **Q2 (nQ2=232)** | **nQ1Q2** | **Jaccard** | **P(Q2\|Q1)** | **P(Q1\|Q2)** |
| --- | --- | --- | --- | --- | --- |
| Patient-led signals (n=99) | GenAI as a low-threshold and always-available point of contact (n=98) | 26 | 0.152 | 26.3% | 26.5% |
| Patient-led signals (n=99) | Standardized and tireless, but relationally thin (n=158) | 41 | 0.190 | 41.4% | 25.9% |
| Patient-led signals (n=99) | Nonjudgmental acceptance as both comfort and clinical hazard (n=69) | 16 | 0.105 | 16.2% | 23.2% |
| Patient-led signals (n=99) | Useful as an adjunct, not acceptable as a replacement (n=47) | 16 | 0.123 | 16.2% | 34.0% |
| Clinician-led signals (n=46) | GenAI as a low-threshold and always-available point of contact (n=98) | 12 | 0.091 | 26.1% | 12.2% |
| Clinician-led signals (n=46) | Standardized and tireless, but relationally thin (n=158) | 19 | 0.103 | 41.3% | 12.0% |
| Clinician-led signals (n=46) | Nonjudgmental acceptance as both comfort and clinical hazard (n=69) | 8 | 0.075 | 17.4% | 11.6% |
| Clinician-led signals (n=46) | Useful as an adjunct, not acceptable as a replacement (n=47) | 11 | 0.134 | 23.9% | 23.4% |
| AI as a relational object (n=66) | GenAI as a low-threshold and always-available point of contact (n=98) | 22 | 0.155 | 33.3% | 22.4% |
| AI as a relational object (n=66) | Standardized and tireless, but relationally thin (n=158) | 29 | 0.149 | 43.9% | 18.4% |
| AI as a relational object (n=66) | Nonjudgmental acceptance as both comfort and clinical hazard (n=69) | 19 | 0.164 | 28.8% | 27.5% |
| AI as a relational object (n=66) | Useful as an adjunct, not acceptable as a replacement (n=47) | 5 | 0.046 | 7.6% | 10.6% |
| AI-mediated changes in the patient-clinician interface (n=60) | GenAI as a low-threshold and always-available point of contact (n=98) | 12 | 0.082 | 20.0% | 12.2% |
| AI-mediated changes in the patient-clinician interface (n=60) | Standardized and tireless, but relationally thin (n=158) | 30 | 0.160 | 50.0% | 19.0% |
| AI-mediated changes in the patient-clinician interface (n=60) | Nonjudgmental acceptance as both comfort and clinical hazard (n=69) | 10 | 0.084 | 16.7% | 14.5% |
| AI-mediated changes in the patient-clinician interface (n=60) | Useful as an adjunct, not acceptable as a replacement (n=47) | 6 | 0.059 | 10.0% | 12.8% |

*Note.* nQ1 and nQ2 indicate the total numbers of respondents who provided interpretable responses to each item. Theme-specific frequencies are shown in parentheses next to each theme label. nQ1Q2 indicates the number of respondents in whom the paired themes co-occurred across Q1 and Q2. Jaccard was calculated as nAB/(nA+nB−nAB). Of these, 144 respondents provided interpretable responses to both Q1 and Q2 and were included in this analysis (pairwise eligible n = 144). Theme-specific frequencies are not mutually exclusive; a respondent may contribute to more than one theme, and theme totals therefore exceed item-level respondent counts.

**Table S2. Cross-question thematic alignments between Q2 and Q3**

| **Q2 (nQ2=232)** | **Q3 (nQ3=220)** | **nQ2Q3** | **Jaccard** | **P(Q3\|Q2)** | **P(Q2\|Q3)** |
| --- | --- | --- | --- | --- | --- |
| GenAI as a low-threshold and always-available point of contact (n=98) | Governance and accountability as prerequisites for adoption (n=130) | 52 | 0.295 | 53.1% | 40.0% |
| GenAI as a low-threshold and always-available point of contact (n=98) | Safety infrastructures for crisis situations and vulnerable populations (n=31) | 14 | 0.122 | 14.3% | 45.2% |
| GenAI as a low-threshold and always-available point of contact (n=98) | Technical reliability and clinical validation before scale-up (n=118) | 54 | 0.333 | 55.1% | 45.8% |
| GenAI as a low-threshold and always-available point of contact (n=98) | Education, supervision, and structural support for responsible use (n=73) | 30 | 0.213 | 30.6% | 41.1% |
| Standardized and tireless, but relationally thin (n=158) | Governance and accountability as prerequisites for adoption (n=130) | 89 | 0.447 | 56.3% | 68.5% |
| Standardized and tireless, but relationally thin (n=158) | Safety infrastructures for crisis situations and vulnerable populations (n=31) | 21 | 0.125 | 13.3% | 67.7% |
| Standardized and tireless, but relationally thin (n=158) | Technical reliability and clinical validation before scale-up (n=118) | 73 | 0.360 | 46.2% | 61.9% |
| Standardized and tireless, but relationally thin (n=158) | Education, supervision, and structural support for responsible use (n=73) | 52 | 0.291 | 32.9% | 71.2% |
| Nonjudgmental acceptance as both comfort and clinical hazard (n=69) | Governance and accountability as prerequisites for adoption (n=130) | 35 | 0.213 | 50.7% | 26.9% |
| Nonjudgmental acceptance as both comfort and clinical hazard (n=69) | Safety infrastructures for crisis situations and vulnerable populations (n=31) | 9 | 0.099 | 13.0% | 29.0% |
| Nonjudgmental acceptance as both comfort and clinical hazard (n=69) | Technical reliability and clinical validation before scale-up (n=118) | 38 | 0.255 | 55.1% | 32.2% |
| Nonjudgmental acceptance as both comfort and clinical hazard (n=69) | Education, supervision, and structural support for responsible use (n=73) | 24 | 0.203 | 34.8% | 32.9% |
| Useful as an adjunct, not acceptable as a replacement (n=47) | Governance and accountability as prerequisites for adoption (n=130) | 29 | 0.196 | 61.7% | 22.3% |
| Useful as an adjunct, not acceptable as a replacement (n=47) | Safety infrastructures for crisis situations and vulnerable populations (n=31) | 5 | 0.068 | 10.6% | 16.1% |
| Useful as an adjunct, not acceptable as a replacement (n=47) | Technical reliability and clinical validation before scale-up (n=118) | 22 | 0.154 | 46.8% | 18.6% |
| Useful as an adjunct, not acceptable as a replacement (n=47) | Education, supervision, and structural support for responsible use (n=73) | 10 | 0.091 | 21.3% | 13.7% |

*Note.* nQ2 and nQ3 indicate the total numbers of respondents who provided interpretable responses to each item. Theme-specific frequencies are shown in parentheses next to each theme label. nQ2Q3 indicates the number of respondents in whom the paired themes co-occurred across Q2 and Q3. Jaccard was calculated as nAB/(nA+nB−nAB). Of these, 210 respondents provided interpretable responses to both Q2 and Q3 and were included in this analysis (pairwise eligible n = 210). Theme-specific frequencies are not mutually exclusive; a respondent may contribute to more than one theme, and theme totals therefore exceed item-level respondent counts.
